# Poster Session I - A186 IMPACT OF TRANSFER TIMING ON LIVER TRANSPLANT OUTCOMES: A SINGLE-CENTER COHORT STUDY

**DOI:** 10.1093/jcag/gwaf042.186

**Published:** 2026-02-13

**Authors:** Y Yao, Y Zhao, E G Kaye, N Sidhu, D Chahal

**Affiliations:** Department of Internal Medicine, University of British Columbia, Vancouver, BC, Canada; School of Medicine, University of British Columbia, Vancouver, BC, Canada; School of Medicine, University of British Columbia, Vancouver, BC, Canada; School of Medicine, University of British Columbia, Vancouver, BC, Canada; Department of Gastroenterology & Hepatology, University of British Columbia, Vancouver, BC, Canada

## Abstract

**Background:**

Liver transplant (LT) wait times have increased due to growing demand without a corresponding rise in organ supply. Longer wait times from listing to LT are associated with higher mortality. However, the impact of transfer timing from an originating hospital to a transplant center for LT evaluation (LTE) remains unclear.

**Aims:**

To compare outcomes between early versus delayed transfer from admission at the originating hospital to admission at a liver transplant center for LTE.

**Methods:**

We conducted a single-center retrospective cohort study of patients transferred from hospitals in British Columbia to Vancouver General Hospital (VGH) for inpatient LTE. Data were collected from chart review using REDCap. Continuous variables were summarized as median [IQR] and compared with Wilcoxon rank-sum tests; categorical variables were compared using chi-square tests. Cox proportional hazards regression was used to assess all-cause mortality. Analyses were performed in RStudio (version 2023.06.0 + 421), with p < 0.05 considered statistically significant.

**Results:**

41 patients were included. Delayed transfer was defined as ≥ 9 days (median transfer time); early transfer as < 9 days. Baseline characteristics, including age, sex, race, Charlson Comorbidity Index, admission MELD score, and admission diagnosis (ACLF vs. acute decompensated cirrhosis vs. non-acute decompensated cirrhosis), did not differ significantly between groups. Median transfer time was 20 days [IQR 25] for delayed transfers and 4 days [IQR 2] for early transfers. Time from VGH admission to listing decision was similar between groups. There were no significant differences in listing outcome (declined vs. listed), death, or receipt of LT. Patients with delayed transfer had a higher risk of death compared to those with early transfer (HR 2.54), but this was not statistically significant (*p* = 0.171).

**Conclusions:**

In this preliminary analysis, delayed transfer was not significantly associated with adverse LT outcomes. The non-significant trend toward higher mortality with delayed transfer may reflect limited sample size. Larger studies are needed to validate these findings.

**Funding Agencies:**

CIHRDr. Daljeet Chahal receives salary or grant support from: Michael Smith Foundation for Health Research (MSFHR) Vancouver Coastal Health Research Institute (VCHRI) Canadian Institutes of Health Research (CIHR) Canadian Donation and Transplantation Research Program (CDTRP) Organ Donation and Transplant Research Foundation of BC

